# Game-based Learning in Eye Health Education - The Developmental Challenges of E-learning Games

**DOI:** 10.12688/f1000research.169156.2

**Published:** 2026-04-07

**Authors:** Srikanth Maseedupalli, Snigdha Snigdha, Kartic Vaidyanathan, Sitaramanjaneyulu Madhukuri, Sreelakshmi Varada, Yamuna Bollam, Shobha Mocherla, Avinash Pathengay, Ruby Kala Prakasam

**Affiliations:** 1 L V Prasad Eye Institute, Hyderabad, India; 2 Lets Play to Learn Company, Chennai, India

**Keywords:** Game-based learning, game development, gamification, eye health education, medical education.

## Abstract

**Purpose:**

Game-based learning enhances eye health education and skill acquisition but poses distinct challenges in developing effective educational games. The present study aims to demonstrate the developmental challenges encountered at various stages of creating e-learning game prototypes for training eye health professionals.

**Methods:**

Structured focus group discussions (FGDs) were conducted involving all the stakeholders involved in game development, the subject experts, and the technical experts. The four phases of FGDs were as follows: i) Ideation and team development; ii) Identifying the competencies and mapping these with cognition; iii) Concept to prototype development; a) Defining questions and data points; b) Accumulating content; and iv) Prototype testing and feedback. The FGDs were audio recorded, the descriptive data were transcribed verbatim, and the data were thematically analysed.

**Results:**

The challenges differed in every phase of the game development. Data on the strengths and weaknesses of the game development were collected via participant recall. Throughout the project, the technical team had to understand the requirements of the subject experts and vice versa. Participants’ understanding of e-learning games varied across the teams. Choosing appropriate topics for game development, identifying and mapping underlying cognition to exit competency, generating data, translating concepts into games, determining the role of communication in maintaining team dynamics, and seeking early feedback from end-users were some of the challenges encountered.

**Conclusion:**

The lessons learned from overcoming procedural challenges in this study may be applied by others who are developing game-based educational tools for eye health education.

## Introduction

Game-based education ^1, 2^ involves crafting products and services through the application of new technology to theoretical learning. Thus, fusing the two fields, technology and education, has given us what is popularly called game-based learning (GBL), which is popular for its high educational value. The blend of game, technology, and learning creates an engaging and immersive educational experience that can enhance cognitive processes, such as memory, attention, and spatial abilities. ^3^

In response to the evolving landscape of technology and educational paradigms, educators are increasingly adopting innovative pedagogical approaches, including the systematic integration of active learning strategies into mainstream curricula. Although GBL, as one of the active learning tools, seems promising for its educational value, the development of effective e-learning games for eye health education poses significant challenges. The design of such games requires a careful balance of educational content and engaging game elements. ^4^ Tan et al identified lacunae with most game models in incorporating learning behaviour into game design and proposed that game designers could leverage pedagogical components such as the story, challenge, goals, and objectives in designing GBL. Addressing this gap, studies ^2, 5– 8^ have proposed guidelines and principles for designing effective educational computer games. These include incorporating clear learning objectives, aligning game mechanics with educational goals, providing adaptive and personalized learning experiences, and leveraging the principles of game design.

The incorporation of game-based learning into eye health education has garnered significant attention in recent years. ^9– 12^ This emerging discipline has been extensively studied for its benefits and pitfalls. ^2, 5, 13^ Researchers have emphasized the potential of educational digital games in contributing to an interactive and dynamic learning process, leading to a clearer and more functional understanding of various scientific concepts and phenomena.

Driven by a commitment to optimize learning outcomes through active learning strategies, a team at a leading eye care centre embarked on developing gamified e-learning modules for their course curriculum. This multi-disciplinary team comprised subject matter experts, educators, administrators, game developers, and design specialists. The team adopted a multidisciplinary approach to develop game prototypes suitable for a course design and a set of learners. The key aspects of the gamification process included our thorough review of the curriculum, mapping learner competencies, data collection for designing e-learning games, the scope of resources, and team collaboration. In this paper, we aim to explore the developmental challenges of creating e-learning games for eye health education and propose strategies to overcome them.

## Methods

Ethical approval for this qualitative study was obtained from the Institutional Review Board of L V Prasad Eye Institute Ethics Committee (IRB Approval No. LEC-BHR-P-05-22-847). Verbal informed consent was obtained from all participants prior to participation, and the study was conducted in accordance with the principles of the Declaration of Helsinki.

### Focus group discussions

Qualitative data were collected from three focus group discussions ^14^ involving all the collaborators and the technical and instructor teams. I want to say, refine accordingly. The study participants were drawn from the game development team. They covered key domains of eye health education. The group consisted of ophthalmologists, optometrists, and nurses with a mean professional experience of 10 years in learning and development. The technical collaborator has substantial experience in game-based learning and in developing gamified interactive pedagogy. A total of 3 FGDs were conducted, and the mean age of participants was 33 ± 5.24 years. The objective of the FGDs was to consolidate different challenges encountered by team members at different stages of game development. Prior to conducting the FGDs, the educator, the technical team representative, and a study moderator connected through a virtual meeting to structure the discussions. The different phases of game development were identified and labelled. ^15^ Further field work for each phase included the formulation of open-ended questions ^15^ by involving the study moderator and game-developer team. Inputs were collected, and further iterations were performed.

**Figure 1.**
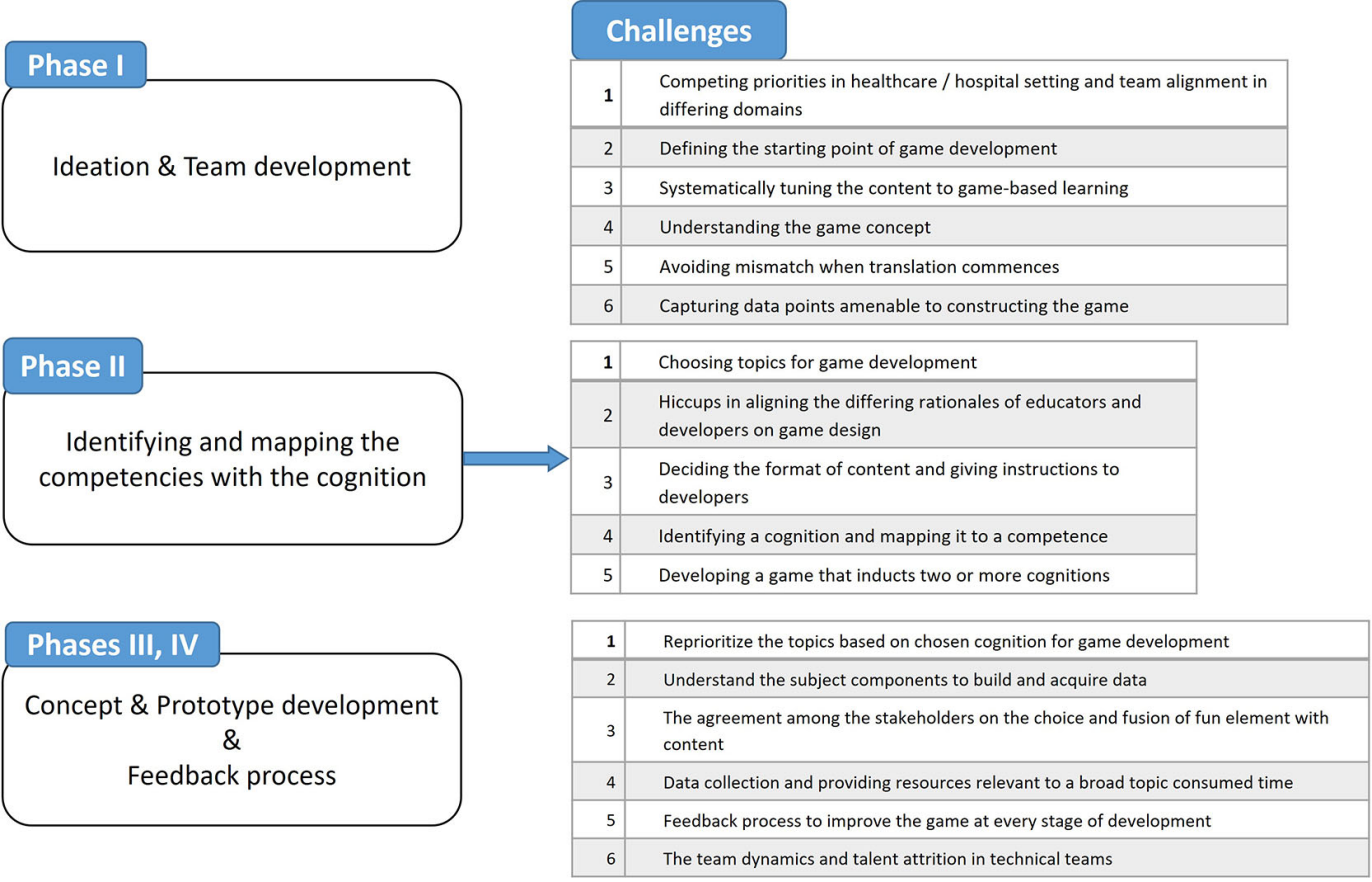
Summary of developmental challenges in game development. Comprehensive list of challenges encountered during each phase of the game development process.

The duration of each focus group discussion was approximately one to two hours. The FGDs were designed and moderated by a third person, the study moderator (RKP), who was not a direct participant in game development. The Zoom Platform for videoconferencing was used to conduct and record the FGDs. Given that the FGDs were conducted and recorded online, a research assistant was not required for notetaking. The study moderator initiated the discussion by asking phase-specific open-ended questions to capture participants’ experience in the selected topic of game development. The audio version was transcribed by the study moderator, and the data were analysed with reference to the phase of the game development identified. We used a deductive thematic analysis approach ^16^ to analyse qualitative data from the focus group discussions. A priori themes were developed based on the study objectives and used to guide the FGD questions. Data were collected under each predefined theme and subsequently analysed to identify key reflections on the challenges encountered at different stages of game development. ^15^ This structured, theme-based approach facilitated systematic comparisons across focus groups. Figure 1 captures the adopted framework and summarizes the developmental challenges.

## Results

### FGD 1 (Phase I) Ideation and team development

Building a successful team requires a strategic blend of passion and expertise. The participants assembled a diverse team comprising internal educators, the subject matter experts with in-depth knowledge of the curriculum and learning objectives, and external technical experts, specifically game developers with the skills essential for e-learning game development. Educators and game developers engaged in team development through collaborative sessions to achieve the following goals:

- Idea Exchange: Brainstorming and refining game concepts to ensure alignment with learning goals.
- Expectation Setting: Establishing clear communication channels and defining roles for each team member.
- Process Rollout: Developing a shared understanding of the development workflow and key milestones.

The formation of a cross-functional team with complementary expertise facilitated effective ideation and laid the foundation for the next project phases. The five challenges encountered during ideation and team development are listed in Figure 1.

### FGD 2 (Phase II) – Identifying and mapping the competencies with the cognition

Traditional teaching involves a set curriculum with lectures, practice, and assessments. This FGD highlighted the potential of game-based learning to improve the educational experience. It emphasised the importance of considering learner motivation, engagement, and knowledge retention in game design. The process of defining learning objectives and matching them with the optimal cognitive process was reflected as a constant endeavour throughout the game development. This allowed team members to identify potential gaps and accordingly adjust the game design.

The collaboration between educators with limited digital experience and the developers was crucial for creating effective game prototypes. Due to limited resources, the team strategically identified specific topics within the curriculum that would benefit students the most from gamification. Furthermore, game objectives were aligned with educational learning goals by mapping trainee exit competencies to relevant cognitive abilities, as illustrated by a sample topic from the Ophthalmic Nursing Assistants course shown in Table 1. This structured mapping process was also applied to the other selected course topics during game development.

**Table 1.** Identification and mapping of competencies to cognitive skills.

Example topic from the ONA * course: Preparing patients for general and specific eye surgery	
1	Exit competency: Ability to prepare a patient for surgery
2	Required Knowledge: Understanding of the step-by-step procedure, including associated dos and dont’s
3	Cognitive skills required: Order memory and decision-making skills
4	Game objective: To impart technical knowledge while concurrently enhancing order memory and decision-making skills

Steps involved in identifying and mapping the competencies to the cognitive skills required for learning a topic, illustrated using an example topic.

* Ophthalmic Nursing Assistants.

### FGD 3 (Phases III & IV): Concept & prototype development

Educators and facilitators initially had limited experience with 2D and text-based teaching tools. The concept of a game-based learning tool on a digital platform was entirely new, requiring collaboration with developers. The team strategically began with simple concepts to build experience and gradually progressed to creating more engaging 3D game prototypes.

This FGD highlighted the importance of creating a balance between educational value and engaging gameplay. It underscored the need for collaboration between educators with limited digital expertise and developers during the game design and development process. This iterative approach, starting with simpler concepts, allowed the team to build their experience and develop more sophisticated 3D prototypes.

i. **Game concept development:** This phase involved brainstorming and refining game concepts that incorporate fun elements to enhance educational value. Based on the chosen concept, this phase focused on accumulating relevant content (text, images, etc.) to populate the game.
ii. **Prototype development:** This iterative process involved:
  a. Creating a basic prototype that focuses on core mechanics and functionality. The main mechanics include point-based scoring, sequence-ordering tasks, decision-making steps, and time-limited responses.
  b. Refining the prototype based on testing and feedback.
  c. The development approach, including the use of structured digital templates during the early phase and progressive movement toward custom-developed prototypes, with the possibility of incorporating 3D features.
  d. The topics addressed include patient preparation for surgery, sterilisation protocols, and clinical workflow in ophthalmic settings.
iii. **Prototype testing and feedback process:** This phase involved conducting user testing with the target audience to gather feedback and ongoingly identify areas for improvement.

### Reflections on challenges

- A minimal understanding of the subjects was essential for building and acquiring data for game development. As a technical team member stated, “The ophthalmic terms were new. For example, ‘sterility’ I understand, but deeper levels of the sterilization process were new.”
- A member of the education team said, “It took us considerable time to understand and attain the threshold level of knowledge about every subject and then build on that.”
- The fusion of fun elements with the content earmarked for learning mandated collaboration between the technical and educator teams.
- Time was initially invested in helping the team of educators learn the technical details and helping the technical team grasp the subject in its entirety. In the process of game development, team collaboration was critical to both data collection and data integration.
- Data collection relevant to a broad topic demanded frequent interactive meetings and follow-up. This called for the creation and sharing of subject-related content and high-quality clinical pictures in structured and special game-specific formats.
- The initial prototypes were subjected to closed feedback, the suggestions were shared with the team of developers, and corrections were implemented.
- Team dynamics and talent attrition in technical teams: The exit of developers from the technical team just when we had attained project efficiency significantly delayed the project. Given the boom in the technology market, engineer retention was a challenge in our short-duration project for developing e-learning games. When an engineer resigned and left the team, it was observed that there was no return on investment because of the high cost of knowledge transfer (KT) made during the training and development of the team.

## Discussion

Our experiences in developing e-games echoed the common challenges ^17^ reported in the literature, including limited technical skills, time constraints, inadequate infrastructure, poor communication, and negative educator attitudes. Several studies have proposed strategic approaches to address these barriers and support effective gamification implementation. ^17– 20^ This section presents our strategic approach to addressing the challenges encountered, along with a proposed framework to guide game developers and educators in effective implementation.

Our journey revealed crucial insights throughout the game development process. Notably, the establishment of a robust user feedback loop emerged as a critical step before finalizing the game. Stakeholders, including content creators who identified key information, educators who defined functional needs, and learners who provided user-centric perspectives, all played vital roles in shaping the game. However, user requirements pertaining to the desired level of complexity, implementation strategies, and feedback mechanisms often remained ambiguous. The development team sometimes gravitated towards advanced functionalities, and initially, readily available game platform templates with “plug-and-play” features were viewed favourably. However, custom-built games that demanded coding expertise presented significant cost and development hurdles. Faculty and educators needed to prioritize concepts requiring gamification and carefully map learning objectives to corresponding cognitive skills.

Gamification impacts the motivation, cognition, emotion, and sociality of players. ^21, 22^ To cultivate an engaging learning experience, we incorporated visible game mechanics like scoring and progress indicators, along with the ability to track player metrics. Notably, we consciously avoided implementing punitive measures such as negative grading for incorrect choices. In retrospect, a more structured approach would have been beneficial. Scoping the project upfront, ensuring alignment between requirements and available resources, and setting realistic goals could have streamlined the development process. Our experience emphasizes the importance of iterative course correction, leading to successful project completion. The early recognition of the value of project scoping and the visualization of final deliverables were key takeaways from this endeavour.

### Strengths of the study

This study represents a carefully revised and peer-reviewed version of our earlier preprint, ^23^ with enhanced clarity, updated analyses, and improved presentation of data. To the best of our knowledge, this research offers a novel exploration of the challenges associated with developing gamified e-learning programs in eye health education. Our findings can serve as a foundation for future research in this domain. As a concluding contribution, we propose a checklist to guide the development journey of gamified learning experiences:

- Module Selection: Identifying specific modules within the curriculum best suited for gamification.
- Content Curation: Defining the essential knowledge required for game development.
- Media Integration: Selecting appropriate images and educational artifacts to enhance the game.
- Platform Choice: Deciding between custom-built platforms or existing game templates.
- Custom Development Challenges: Assessing the complexities involved in building bespoke games.
- Resource Evaluation: Investigating the availability of technical expertise and addressing potential market challenges.

By implementing this framework, educators and developers can embark on a more efficient and well-defined path when creating engaging and effective gamified e-learning experiences.

### Limitations of the study

Although online FGDs present inherent limitations, including reduced non-verbal cues, weaker group dynamics, and potential distractions, these were partially mitigated in this study. Participants were provided with stable internet access, instructed to keep cameras on, and briefed on the use of the platform. Pre-designed discussion themes were employed to maintain focus and minimise topic deviation. The online format also facilitated the inclusion of participants from diverse locations, eliminating the need for travel. However, this study involved developers from a single institute, and the variations in institutional practice, cultural context, resource availability, and local industry standards may limit the generalisability of the findings. The inter-rater reliability was not assessed because the FGD data were analysed by a single researcher. Furthermore, the proposed six-item checklist should be considered a preliminary guide that will need prospective validation in future game development work.

## Conclusion

Gamification facilitates cognitive learning, serving the broader purpose of conventional teaching and assessment methods. Setting expectations about the use of the final product could help teams make the e-learning game user-friendly. Given that gaming is an active learning tool, gamification can only supplement the traditional modes of instruction and learning. We fulfilled the objective of our project by bringing in fun, making it interactive, and developing a failsafe learning approach to creating games. Given the variety of games available, realistic selection from a predesigned game platform may prevent over engineering to match various levels of user learning. Finally, in medical education, sophistication in digital gaming is best avoided in the interest of learning per se. The two aims of participating in games, achieving players’ focused attention and their playing with great team spirit, must be served well in gamification and game-based learning.

## Data Availability

The study contains the following data: The underlying data (supplementary material 1; https://doi.org/10.6084/m9.figshare.29880998.v2 ^15^) has been deposited in Figshare: Deductive thematic analysis approach: Pre-defined themes, Guiding questions for FGDs, and Key reflections on the challenges encountered at different stages of game development. The extended data (supplementary material 2; https://doi.org/10.6084/m9.figshare.29880686.v2 ^14^) has been deposited in Figshare: Focus Group Discussions Transcripts. Data are available under the terms of the Creative Commons Attribution 4.0 International license (CC-BY 4.0).
