# Supplementary material for: Game-based Learning in Eye Health Education - The Developmental Challenges of E-learning Games
Source: F1000Res. 2026 Apr 7;14:1390. Originally published 2025 Dec 10. [Version 2] doi: 10.12688/f1000research.169156.2 (PMC13176830; doi:10.12688/f1000research.169156.2)
Supplement: Peer review history

# Reviewer report: Reviewer response for version 2

**DOI:** 10.5256/f1000research.197595.r474130

**Published:** 2026-05-20

**Authors:** Varsha Shridhar

**Affiliations:** 1 Molecular Solutions Care Health LLP, Bengaluru, Karnataka, India

The article presents a description of a multistakeholder consultation to develop gamified teaching modules for ophthalmology. The authors have focussed on one aspect of this process: that of bringing together this multistakeholder collaboration and the challenges faced during the process of development of the gamified educational module due to differences in priorities, desired outcomes and backgrounds. However, the intended audiences & desired outcomes for the learning/ teaching modules and actual outcomes of the development process are not clear. The depth of information provided in the figure and table could be improved.

 Specific feedback points:

 a) What were the themes that emerged from the FGDs? While the challenges faced are appreciated, it would have been useful to see the differences in perspectives and priorities of the various stakeholder groups (practicing clinicians, educators, game developers etc) from the transcript analyses that led to the list of challenges in Fig 1 (see comments below on Reflections on Challenges section). This will enhance the list from a fairly standard list of challenges to something more specific, nuanced and useful in this specific context of ophthalmologic education.

 b) Table 1 maps some of the competency domains for ONAs selected for gamification. While useful, this might have been improved by mapping all the required domains for ONAs and highlighting which ones were chosen for gamification and why. Another way to improve depth of information might have been to include another example of a target population.

 c) Reflections on Challenges section: The quotations from transcripts were useful. Would suggest mapping such quotations to the challenges identified in Fig 1. Some statements in this section are less about reflections and more about activities better suited to the Methods section, such as  “The initial prototypes were subjected to closed feedback, the suggestions were shared with the team of developers, and corrections were implemented”

 d) Finally, there was no mention of the product that emerged from this process: what was the gamified output? What was the feedback for the same from all the stakeholders? 

 e) Other thoughts:

 - “Eye health education” in the title: makes it sound like the audience is the lay population/ patient population. Instead, it actually seems like the authors are interested in developing modules for clinicians, be they nurses or doctors or other healthcare providers. So, would suggest “Ophthalmology education” to highlight this distinction.

- Under Methods, FGD section, 2nd line has a random sentence which needs to be deleted:  “I want to say, refine accordingly”
- The article is actually about the process and challenges of multistakeholder/ interdisciplinary collaborations, using gamified ophthalmological learning modules as an example. It is less about game-based learning per se. Would suggest flipping the title phrases to underscore this.

Is the work clearly and accurately presented and does it cite the current literature?

Yes

If applicable, is the statistical analysis and its interpretation appropriate?

Not applicable

Are all the source data underlying the results available to ensure full reproducibility?

Yes

Is the study design appropriate and is the work technically sound?

Yes

Are the conclusions drawn adequately supported by the results?

Partly

Are sufficient details of methods and analysis provided to allow replication by others?

Partly

Reviewer Expertise:

NA

I confirm that I have read this submission and believe that I have an appropriate level of expertise to confirm that it is of an acceptable scientific standard, however I have significant reservations, as outlined above.

# Reviewer report: Reviewer response for version 2

**DOI:** 10.5256/f1000research.197595.r478681

**Published:** 2026-05-14

**Authors:** Andreas Marougkas

**Affiliations:** 1 University of West Attica, Egaleo, Greece

The methodology presented in the paper does not sufficiently account for the learners/end users' perspective, which is crucial to this particular topic and type of research. Not including the actual gamers in research on game-based learning undermines the value and significance of the results.

 There is no evidence of improved learning outcomes, increased engagement, better retention of information, development of particular skills, and enhanced performance due to the developed games. In fact, there is nothing but assumptions that these games have educational value.

 The qualitative research methodology does not seem to be properly employed by the authors, since it includes only one coder, does not consider inter-rater reliability, member checking, and triangulation.

 The proposed checklist is incomplete and not validated enough, being based merely on the experience in one institution. The authors need to specify this point to avoid misinterpretations of their findings.

 Moreover, the manuscript needs further editing. "I want to say, refine accordingly" is not an appropriate phrase that would be found in the scholarly article.

Is the work clearly and accurately presented and does it cite the current literature?

Partly

If applicable, is the statistical analysis and its interpretation appropriate?

Not applicable

Are all the source data underlying the results available to ensure full reproducibility?

Yes

Is the study design appropriate and is the work technically sound?

Partly

Are the conclusions drawn adequately supported by the results?

Partly

Are sufficient details of methods and analysis provided to allow replication by others?

Partly

Reviewer Expertise:

Personalization and Gamification in Educational Virtual Reality, Game-Based Learning, Immersive Learning Environments, Educational Technology, Technology-Enhanced Learning, Human–Computer Interaction in Education, VR-Based Training and Learning Systems

I confirm that I have read this submission and believe that I have an appropriate level of expertise to confirm that it is of an acceptable scientific standard, however I have significant reservations, as outlined above.

# Reviewer report: Reviewer response for version 1

**DOI:** 10.5256/f1000research.186443.r445590

**Published:** 2026-01-23

**Authors:** Mohammed Kadri

**Affiliations:** 1 Sidi Mohamed Ben Abdellah University, El Mehraz, Fez, Morocco

This manuscript presents a qualitative study documenting the challenges encountered during the development of gamified e-learning modules for eye health education at L V Prasad Eye Institute, India. The multidisciplinary team of educators, subject matter experts, game developers, and administrators conducted three focus group discussions to systematically capture challenges across four phases: (1) ideation and team development, (2) competency mapping with cognition, (3) concept to prototype development, and (4) prototype testing and feedback.

 Key challenges identified include: cross-disciplinary communication barriers between technical and domain experts, topic selection for gamification, mapping learning competencies to cognitive skills, content generation in game-specific formats, and talent attrition in technical teams. The authors propose a six-item checklist to guide future developers of gamified learning experiences.

**Strengths**

1. **Addresses an underexplored area**: Game-based learning specifically for eye health education is rarely documented in the literature.
2. **Multidisciplinary perspective**: The inclusion of both educators and developers provides balanced insights into the challenges from multiple viewpoints.
3. **Open data availability**: The authors exemplify good research practice by making FGD transcripts and analysis frameworks publicly available via Figshare.
4. **Practical framework**: The proposed six-item checklist provides actionable guidance for future developers.
5. **Ethical rigor**: Ethics approval was obtained, and the study followed the Declaration of Helsinki.
6. **Transparent methodology**: The use of deductive thematic analysis with clearly stated a priori themes enhances reproducibility.

**Major Issues to Address**

1. **Lack of learner perspective**: The study captures developer and educator challenges but does not include the end-user (learner) perspective. Consider conducting additional FGDs or surveys with students who used the developed games to provide a complete picture.
2. **Framework validation**: The proposed six-item checklist is based on a single development experience. The authors should either:
  - Acknowledge this as a preliminary framework requiring validation, OR
  - Test the checklist prospectively in a subsequent development project
3. **Generalizability limitations**: The findings are from one institution in one country. The authors should explicitly discuss how cultural, institutional, and resource contexts may affect transferability of findings.
4. **Learning outcomes**: There is no assessment of whether the developed games actually achieved their educational objectives. At minimum, preliminary evaluation data would strengthen the manuscript.

**Minor Issues**

1. **Game prototype descriptions**: Please provide more detail about the actual games developed (genre, mechanics, platforms, topics covered).
2. **Participant demographics**: Consider reporting additional relevant characteristics (e.g., years of experience, roles in more detail).
3. **Inter-rater reliability**: If multiple coders analyzed the transcripts, please report reliability metrics. If single coder, please acknowledge this limitation.
4. **Figure 1 quality**: The figure summarizing developmental challenges is referenced but ensure it is clearly legible in the published version.
5. **Table 1**: The example of competency mapping is helpful; consider expanding this with additional examples from different course topics.

Future Research Directions and Advisory Suggestions

 The manuscript opens important avenues for future research that the authors should consider addressing in the Discussion section. Based on the challenges identified, particularly around prototype testing, team collaboration, and knowledge transfer, the following research directions would significantly advance this field:

**1. Comparison with Immersive Technologies (VR/AR)**

 The current study focuses on 2D and text-based game prototypes, progressing to 3D elements. A natural extension would be to **compare the effectiveness of traditional e-learning games with immersive Virtual Reality (VR) and Augmented Reality (AR) alternatives** for eye health education.

**Why this matters:**

- VR/AR can provide spatial understanding of anatomical structures (e.g., the eye's 3D anatomy) that 2D games cannot replicate
- Immersive technologies have demonstrated enhanced engagement and presence in medical education contexts
- The challenges documented in this study (content translation, prototype testing) may differ significantly for VR/AR development

**2. Integration of Artificial Intelligence (AI)**

 The authors discuss challenges in content translation and personalized learning experiences. **Integrating AI-based guidance and adaptive learning systems** could address several challenges identified in Phase III (concept to prototype development).

**Why this matters:**

- AI can provide personalized learning pathways based on individual learner performance
- Generative AI assistants can reduce cognitive load during complex medical content exploration
- AI can automate aspects of content generation and adaptation, addressing team workload issues

**3. Collaborative and Social Learning Features**

 The manuscript emphasizes team collaboration challenges between developers and educators. Future game prototypes should explore **collaborative learning features** that enable social presence and peer interaction, which are particularly important for professional training.

**Why this matters:**

- Healthcare education often benefits from social learning and peer discussion
- Collaborative features can enhance knowledge construction through dialogue
- Social presence has been shown to improve engagement and motivation in distance learning

**4. Longitudinal Knowledge Retention Assessment**

 The study acknowledges the lack of learning outcome assessment. Future research should incorporate **longitudinal retention testing** with delayed post-tests to evaluate whether game-based learning produces durable knowledge gains.

**Why this matters:**

- Short-term learning gains may not translate to long-term retention
- Medical professionals need sustained knowledge for clinical practice
- Understanding retention patterns can inform game design decisions about repetition and reinforcement.

1- The manuscript presents a well-structured qualitative study documenting the developmental challenges of e-learning games for eye health education. The four-phase framework (ideation, competency mapping, prototype development, testing) provides a clear organizational structure. The literature review adequately covers game-based learning, gamification principles, and existing applications in medical education. The references are current and relevant, with most citations from 2020-2024.

 2- The qualitative research design using focus group discussions (FGDs) is appropriate for the exploratory nature of this research. The deductive thematic analysis approach with a priori themes is suitable for investigating predefined developmental phases.

 Areas for improvement:

 - The study would benefit from inter-rater reliability assessment for the thematic coding process

 - Member checking with participants to validate findings would strengthen credibility

 - The study lacks learner/end-user perspective - only developers and educators were included

 - No triangulation with other data sources (e.g., project documents, observation notes)

 3- The authors have commendably made raw data available through Figshare (FGD transcripts and thematic analysis frameworks), which supports reproducibility.

 Missing details for full replication:

 - Specific characteristics of the developed game prototypes are not described

 - The topics selected for gamification are mentioned in Table 1 but not fully elaborated

 - The exact decision-making process for theme identification could be more explicit

 - Participant selection criteria beyond team membership are unclear

 4- This is a qualitative study. The only quantitative data reported is participant demographics (mean age 33 ± 5.24 years), which is appropriately presented.

 5- The authors have made underlying data available through Figshare:

 - Supplementary Material 1: Deductive thematic analysis approach, pre-defined themes, guiding questions, and key reflections

 - Supplementary Material 2: Focus Group Discussion transcripts

 Data are available under Creative Commons Attribution 4.0 International license (CC-BY 4.0). This adherence to FAIR data principles is commendable.

 6- The conclusions about developmental challenges are well-supported by the qualitative findings from FGDs. The proposed six-item checklist is a practical contribution.

 However:

 - The generalizability of findings from a single institution is limited

 - The proposed checklist has not been empirically validated

 - The claim that "lessons learned... may be applied by others" would be strengthened by testing the framework in different contexts

 - No assessment of whether the developed games actually improved learning outcomes

Is the work clearly and accurately presented and does it cite the current literature?

Yes

If applicable, is the statistical analysis and its interpretation appropriate?

Not applicable

Are all the source data underlying the results available to ensure full reproducibility?

Yes

Is the study design appropriate and is the work technically sound?

Partly

Are the conclusions drawn adequately supported by the results?

Partly

Are sufficient details of methods and analysis provided to allow replication by others?

Partly

Reviewer Expertise:

Educational Technology, Immersive Learning (VR/AR), Game-based Learning, Medical Education Technology, HCI, AI-Enhanced Pedagogy

I confirm that I have read this submission and believe that I have an appropriate level of expertise to confirm that it is of an acceptable scientific standard, however I have significant reservations, as outlined above.

## References

[1] : IVAL: Immersive Virtual Anatomy Laboratory for enhancing medical education based on virtual reality and serious games, design, implementation, and evaluation. *Entertainment Computing* .2024;49: 10.1016/j.entcom.2023.100624 10.1016/j.entcom.2023.100624

[2] : C-IVAL: A Longitudinal Study of Knowledge Retention and Technology Acceptance in Collaborative Virtual Reality-Based Medical Education. *IEEE Access* .2025;13: 10.1109/ACCESS.2024.3523860 16055-16071 10.1109/ACCESS.2024.3523860

# Response

**Published:** 2026-03-10

**Authors:** Ruby Kala Prakasam

**Affiliations:** L V Prasad Eye Institute, India

**Major Issues to Address**

1. **Lack of learner perspective:** The study captures developer and educator challenges but does not include the end-user (learner) perspective. Consider conducting additional FGDs or surveys with students who used the developed games to provide a complete picture.

**Authors' Response:** Thank you for this important comment. The authors believe the end-user perspective is an important aspect to be studied, which was beyond the scope of the present study.  We will incorporate the end-user perspective in our follow-up study on game-based learning. 

1. **Framework validation:** The proposed six-item checklist is based on a single development experience. The authors should either:
  - Acknowledge this as a preliminary framework requiring validation, OR
  - Test the checklist prospectively in a subsequent development project

**Authors' Response:** Thank you for your valuable comments. Authors also acknowledge that the proposed six-item checklist is a preliminary framework that requires prospective validation as part of our subsequent game development project. This statement has now been included in the manuscript under the section ‘limitations of the study’.

  

1. **Generalizability limitations:** The findings are from one institution in one country. The authors should explicitly discuss how cultural, institutional, and resource contexts may affect the transferability of findings.

**Authors' Response**: Thank you for this important suggestion. We have now included this information on the generalizability of the study from a single institute as a potential limitation of the study  

1. **Learning outcomes:** There is no assessment of whether the developed games actually achieved their educational objectives. At a minimum, preliminary evaluation data would strengthen the manuscript.

**Authors' Response**: Thank you for your valuable suggestion. This study was primarily designed to capture educators' perceived challenges during different stages of the game development process. However, we fully agree with your point. As a follow-up, we are planning a study to assess the end-user perspective of the game and evaluate its educational outcomes. This has now been acknowledged in the manuscript under the ‘Limitations of the Study’ section.

**Minor Issues**

1. Game prototype descriptions: Please provide more detail about the actual games developed (genre, mechanics, platforms, topics covered).

**Response:** We appreciate this important suggestion. We have now added a detailed description of the developed game prototypes in the Results. Specifically, we have clarified:

- The genre of the games (2D and 3D interactive modules).
- The core mechanics, including scoring systems, order-based sequencing tasks, decision-making pathways, and timed responses.
- The platform approach, including the use of structured digital templates in early stages and gradual progression to custom-built prototypes.
- The topics covered include patient preparation for surgery, sterilization protocols, and procedural workflow in ophthalmic settings.

 

1. Participant demographics: Consider reporting additional relevant characteristics (e.g., years of experience, roles in more detail).

Thank you for your valuable comment. We have included relevant characteristics in the methods section of the manuscript

 All participants in this study were involved in game development and represented several domains of eye health education on a full-time basis. This group is a comprehensive representation of several cadres of eye health professionals, Ophthalmologists, Optometrists, and Nurses with an average of 10 years of experience in learning and development. The technical collaborator is a pioneer in game-based learning and a specialist in gamified interactive pedagogy.

  

1. Inter-rater reliability: If multiple coders analyzed the transcripts, please report reliability metrics. If single coder, please acknowledge this limitation.

**Authors' Response:** Thank you for this important comment. The FGD transcripts were analysed by a single coder. As suggested, we have now added this information to the limitations section of the manuscript

1. Figure 1 quality: The figure summarizing developmental challenges is referenced but ensure it is clearly legible in the published version.

**Authors' Response:** Thank you. As suggested, we have replaced Figure 1 with a legible text.

1. Table 1: The example of competency mapping is helpful; consider expanding this with additional examples from different course topics.

**Authors' Response:** We thank the reviewer for this thoughtful suggestion. Table 1 was intentionally designed to provide a single, clear illustrative example from the Ophthalmic Nursing Assistant (ONA) course to demonstrate the structured process of mapping exit competencies to cognitive skills and corresponding game objectives. The purpose of the table is to exemplify the methodological approach rather than to present topic-wise results. In methodological manuscripts, presenting one representative example is often preferred to maintain conceptual clarity and readability. Including multiple examples across topics could increase complexity and potentially reduce the ease with which readers understand the mapping framework.

 However, to improve transparency, we have now clarified in the manuscript that "the same structured mapping process was systematically applied across other selected course topics during development." We appreciate the reviewer’s insight, which has helped us make this methodological intent more explicit".

 Future Research Directions and Advisory Suggestions

 The manuscript opens important avenues for future research that the authors should consider addressing in the Discussion section. Based on the challenges identified, particularly around prototype testing, team collaboration, and knowledge transfer, the following research directions would significantly advance this field:

**Authors' Response:** We thank you for your suggestion regarding future research directions. The points mentioned are well taken, and we will consider them in our future work.

# Reviewer report: Reviewer response for version 1

**DOI:** 10.5256/f1000research.186443.r441450

**Published:** 2026-01-02

**Authors:** Chen Zhi Xiong

**Affiliations:** 1 National University of Singapore, Singapore, Singapore

The methodologies are sound. While the lessons learned are not entirely novel, they are sufficiently detailed and remain useful to eye health professionals' context. The article documents real-world development challenges. This aligns well with F1000's scope. The article is also clearly written. A suggestion for improvement may be to include learners/players of the game in the FGDs, so that the end users' perspectives can be captured to enhance future development process.

Is the work clearly and accurately presented and does it cite the current literature?

Yes

If applicable, is the statistical analysis and its interpretation appropriate?

Not applicable

Are all the source data underlying the results available to ensure full reproducibility?

Yes

Is the study design appropriate and is the work technically sound?

Yes

Are the conclusions drawn adequately supported by the results?

Yes

Are sufficient details of methods and analysis provided to allow replication by others?

Yes

Reviewer Expertise:

Pediatric solid tumors, health professions education

I confirm that I have read this submission and believe that I have an appropriate level of expertise to confirm that it is of an acceptable scientific standard.

# Response

**Published:** 2026-01-06

**Authors:** Ruby Kala Prakasam

**Affiliations:** L V Prasad Eye Institute, India

Dr.Zhi, Thank you for your valuable suggestion. We will incorporate the end-user perspective in our follow-up study on game-based learning.
